# Valorization of Sour Buttermilk (A Potential Waste Stream): Conversion to Powder Employing Reverse Osmosis and Spray Drying

**DOI:** 10.3390/membranes13090799

**Published:** 2023-09-17

**Authors:** Subhadip Manik, Ganga Sahay Meena, Ashish Kumar Singh, Yogesh Khetra, Richa Singh, Sumit Arora, Raghu H. Vishweswaraiah

**Affiliations:** 1 Dairy Technology Division, ICAR-National Dairy Research Institute, Karnal 132001, Haryana, Indiayogesh.khetra@icar.gov.in (Y.K.); 2 Dairy Chemistry Division, ICAR-National Dairy Research Institute, Karnal 132001, Haryana, India; 3 Dairy Microbiology Division, ICAR-National Dairy Research Institute, Karnal 132001, Haryana, India

**Keywords:** sour buttermilk, reverse osmosis, domestic waste stream, valorization, dairy ingredient, spray drying, amino acid and fatty acid profiling

## Abstract

Reverse osmosis (RO) is known for the economic dewatering of dairy streams without any change in phase. At the household level, surplus milk is fermented and churned to obtain butter, which is subsequently heated to obtain clarified milk fat (*ghee*). The production of 1 kg *ghee* generates 15–20 kg sour buttermilk (SBM) as a by-product that is mostly drained. This causes a loss of milk solids and environmental pollution. The processing, preservation and valorization of SBM are quite challenging because of its low total solids (TS) and pH, poor heat stability and limited shelf life. This investigation aimed to transform SBM into a novel dried dairy ingredient. SBM was thermized, filtered, defatted and concentrated at 35 ± 1 °C, employing RO up to 3.62× (12.86%). The RO concentrate was subsequently converted into sour buttermilk powder (SBMP) by employing spray drying. SBMP was further characterized for its physicochemical, reconstitution and functional properties; rheological and morphological characteristics; and amino acid and fatty acid profiling, along with FTIR and XRD spectra. SBMP was “instant soluble-3 s” and exhibited excellent emulsion stability (80.70%), water binding capacity (4.34 g/g of protein), flowability (28.36°) and antioxidant properties. In nutshell, a process was developed for the valorization of sour buttermilk to a novel dairy ingredient by employing reverse osmosis and a spray-drying process.

## 1. Introduction

The reduction of food loss and waste is extremely essential from a global perspective, as the number of people affected by hunger has gradually increased since the year 2014, and millions of tons of wholesome food is lost or wasted everyday worldwide. Globally, around 14% of food produced is lost between harvest and retail, and 17% of total food production is wasted (i.e., 11% in households, 5% in food service and 2% in retail) [1]. The dairy industry plays a key role in serving the food demands of people but also causes significant pollution, as it generates a significant amount of effluent, whose efficient treatment is mandatory prior to its disposal into the environment. Around 4–11 million tons of dairy waste residue is produced each year worldwide in the form of solid waste and effluents [2,3]. Furthermore, every volume of milk that is processed generates an effluent to the tune of 1–3 times. It accounts for an annual waste of 3.739–11.217 million cubic meters [3,4].

The unorganized sector in India produces fermented milk products, consuming 14% of the nation’s total milk production [5]. This sector regularly produces *ghee* to preserve the milk fat, using an indigenous process consisting of the fermentation of whole milk, followed by its churning. In the Indian subcontinent, the production of 1 kg of *ghee* also generates 15–20 kg of sour buttermilk (SBM) [6,7]. Usually, SBM is brownish in color, as the milk is subjected to prolonged heating prior to its inoculation with undefined starter culture, causing uncontrolled fermentation. It generally contains 3.8% TS, 0.8% fat, 1.29% protein, 1.2% lactose, 0.44% lactic acid and 0.4% ash [8]. It contains large size curd particles and is characterized by a nonhomogeneous consistency (prone to settling and accumulation of watery portion on the top) due to its higher acidity. Curd deposition is frequently observed when kept undisturbed [9,10]. The key factors posing numerous challenges in its utilization include its limited shelf life, high acidity, low heat stability, huge bulk with low TS content and lack of proper collection and processing system [11]. These are the probable reasons for the non-inclusion of SBM in mainstream dairy processing. Therefore, it is drained into the environment.

RO is termed as a concentration or dewatering membrane process. It can selectively separate solutes with a molecular weight of more than 150 Daltons [12]. Recent developments improved the functionality of RO membranes, endowing them with characteristics such as acid resistance, high retention, antifouling and ultralow pressure [13]. Liquid dairy streams such as milk, whey and buttermilk can be efficiently concentrated without any compositional change (i.e., ratio of milk constituents remains same) by the RO process [14]. The feed handling flexibility, operational simplicity and affordable cost make it suitable for use at the farm-to-industrial scale. Sweet cream buttermilk (SCBM) was successfully concentrated by employing RO from its initial 8.2% total solids to 21% after a 2.56-fold concentration [15].

Spray drying is rapid and suits large-scale production. Drying prevents product deterioration during storage by reducing the water activity. Additionally, it lowers the transportation cost and simplifies its application in a variety of food formulations. The drying process brings numerous structural and physicochemical changes that impact the handling and rehydration characteristics of dairy powders and their shelf life [16]. Tamime et al. [17] recommended the pumping of acidified buttermilk concentrate into a spray drier at 43 °C, with a recommended inlet temperature between 175 to 195 °C and a low outlet air temperature to control powder discoloration.

For the inclusion of SBM into the main dairy stream, the required scientific method is not available. Therefore, the current investigation aimed to develop a method for the processing, preservation and valorization of SBM, along with detailed characterization.

## 2. Materials and Methods

### 2.1. Procurement, Analysis, Pretreatments and Concentration of SBM

**Figure 1:**
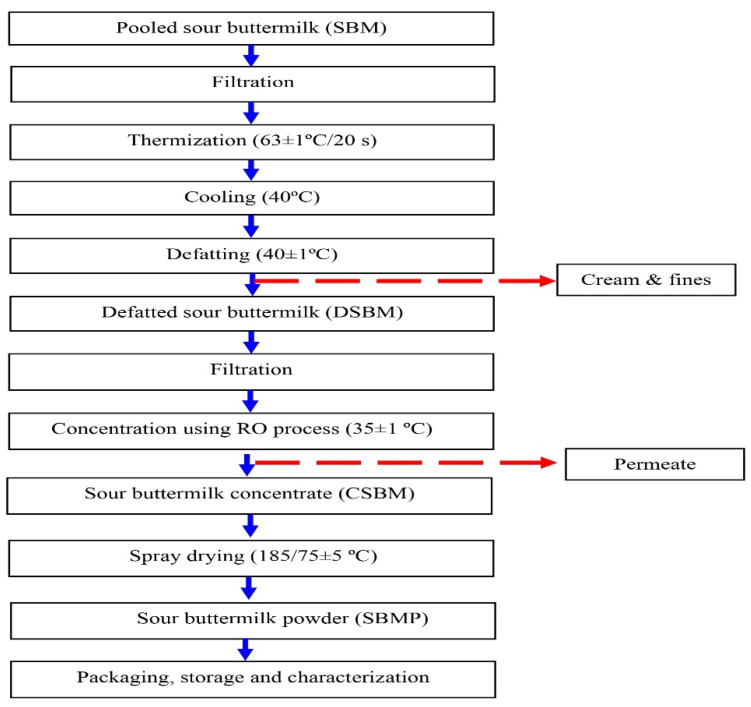
Production process of sour buttermilk powder (SBMP).

Analytical-grade chemicals procured from Sigma Aldrich (Bengaluru, India) and HiMedia Laboratories (Mumbai, India) were used in this investigation. For each trial, fresh SBM samples produced via the traditional method were procured from the countryside farmers (29.6857° N, 76.9905° E; Karnal, India) in the early morning. Thereafter, SBM samples were pooled and then thermized (63 °C/20 s), cooled (40 ± 1 °C) and subsequently defatted using centrifugal separation (Model: Kamdhenu KD-600; Make: Sinhal Metal India Pvt. Ltd., New Delhi, India). The obtained defatted sour buttermilk (DSBM) was filtered through a muslin cloth and concentrated by employing the RO process, maintaining a 30 ± 5 bar pressure and 35 ± 1 °C temperature to obtain sour buttermilk concentrate (CSBM) with maximum possible TS (Figure 1). RO plant was supplied by Peterson Candy International Ltd., Reading, UK. A total membrane area of 0.9 m^2^ (polyamide, AFC 99) was installed in this plant (length—1500 mm; height—800 mm; depth—700 mm; weight—70 kg). The plant was equipped with Type B1 tubular module of 1.2 m in length. It also had a shroud and heat exchanger (length—0.6 m) made of SS 316. A triple plunger pump (contact surface-SS, 4 kW motor; flow, 22 liters/min), which can generate 70 bar pressure, was used. The reported flux range of this plant was 15–60 LMH (L/m^2^/h). Its hold-up volume was 6.50 L. The plant can be operated at a maximum of 70 bar of pressure and can withstand 3–11 pH and 70 °C. 

**Figure 2:**
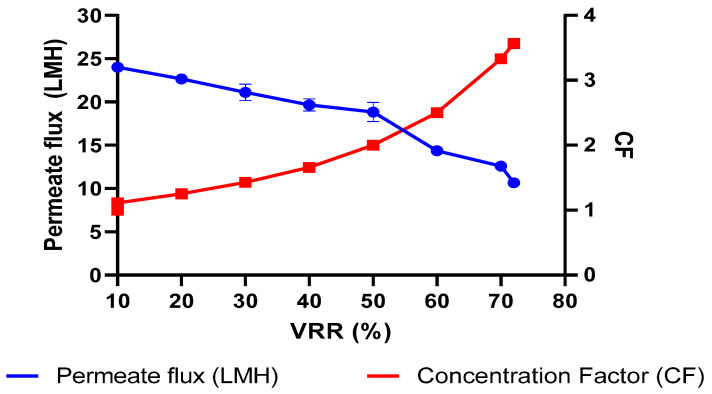
Change in permeate flux as a function of percent volume reduction ratio and concentration factor.

The change in permeate flux was recorded and expressed as a function of percent VRR and concentration factor (*CF*), as shown in Figure 2. The flux mean (*FM*) was calculated from initial flux (*IF*) and final flux (*FF*) values using following formula earlier described by St-Gelais et al. [18].

$$FM=FF+\left[0.33\times \left(IF-FF\right)\right]=10.67+[0.33\times \left(24-10.67\right)]=15.07\;LMH$$

### 2.2. Spray Drying of CSBM

A single-stage spray drier (Jektron Pvt. Ltd., Pune, India) equipped with rotary atomizer was used for the spray drying (185/75 ± 5 °C of RO concentrate to obtain SBMP powder. This non-agglomerated SBMP was immediately packed and sealed in metalized polyester-LDPE laminates, which were stored at 4 ± 1 °C till further analysis. Sour buttermilk powder (SBMP) was prepared (Figure 1) and analyzed in triplicate

### 2.3. Analysis of Chemical Composition and Determination of Physical Properties of Different Buttermilk Samples and SBMP

Total solids (TS), protein, ash and fat contents of SBM, DSBM, CSBM and SBMP samples were determined by adopting solids in milk and powder (AOAC official method of analysis 925.23 and 927.05), Kjeldhal (AOAC official method of analysis 991.20), ash in milk and powder (AOAC official method of analysis 945.46, 930.30) and Mojonnier (AOAC official method of analysis 989.05) methods as per AOAC [19], while the Lane Eynon method [20] was used to estimate their lactose content. The free fat of SBMP was determined by adopting the method reported by Hall and Hedrick [21]. Hydroxymethylfurfural and 2-thiobarbituric acid were measured by adopting the method described by Keeney and Bassette [22] and Hegenauer et al. [23], respectively. A calibrated pH probe (Eutech, Cyberscan 1100, Thermo Scientific, Waltham, MA, USA) was used to measure the pH of different buttermilk samples and reconstituted 10% (*w/v*) SBMP solution, while their acidity values were determined as per the IS: SP:18 method [24]. The ζ-potential of these samples was measured by adopting the method described by Mahadev and Meena [25], using Zetasizer Nano ZS, (Malvern, UK) at 25 °C. The color values of the above samples were recorded using Hunter Lab model color Flex^®^ (Mini-Scan XE plus, Hunter Associates Laboratory Inc., Reston, VA, USA). The water activity (a_w_) of SBMP was determined in Aqua Lab (Model Series 3 TE; supplied by M/s Decagon Devices, Pullman, WA, USA).

### 2.4. Determination of Bulk and Flow Properties of SBMP

The interstitial air content (IAC), occluded air content (OAC) and particle density (PD) of SBMP were determined as per the Niro Atomizer [26] method, while the loose bulk density (LBD), packed or tapped density (PBD), porosity and flowability (in terms of angle of repose, θ°) were determined by adopting the methods reported by Sjollema [27]. The Hausner ratio (HR = PBD/LBD) and Carr index (CI) = [(PBD − LBD) × 100/LBD] were calculated using LBD and PBD values of SBMP.

### 2.5. Determination of Reconstitution and Functional Properties of SBMP

The method described by Muers and House [28] and American Dry Milk Institute [29] were used to determine the wettability and dispersibility of SBMP, respectively. The solubility index for SBMP was measured by adopting the method reported by Schuck et al. [30]. Briefly, 10 g SBMP was reconstituted and mixed for 90 s in 100 mL water at 24 °C, using a solubility index mixer (LABINCO L295, Breda, The Netherlands), and then centrifuged in 50 mL centrifuged tubes at 160× *g* for 10 min. The sediment volume obtained in mL after the second centrifugation was defined as the *insolubility index*. The *solubility index* (*SI*, %) was calculated from the following equation, as reported by Schuck et al. [30].

$$Solubility\;index\;\left(SI\right),\;\%=100-(2\times insolubility\;index)$$

The water binding capacity (WBC), oil binding capacity, emulsification capacity, emulsification stability, foam stability and capacity of SBMP were determined by adopting the method reported by Shilpashree et al. [31]. The buffering capacity of SBMP (0.5% protein solution) was determined by adopting the method reported by Mann and Malik [32].

### 2.6. Determination of Rheological Properties of SBM, DSBM, CSBM and Reconstituted SBMP Samples

The flow curve of SBM, DSBM, CSBM and reconstituted (10% *w*/*v*) SBMP samples was determined using a rheometer (model; MCR52; make: Anton Paar, Graz, Austria) with an attached cup and bob (CC27) probe between 0 to 500 s^−1^ shear rate at 20 °C. Change in apparent viscosity (ƞ_Appa_. mPa.s) of these samples as a function of rise (5 °C min^−1^) in temperature was also recorded at a constant (100 s^−1^) shear rate, as reported by Patil et al. [33].

### 2.7. Analysis of Particle Size Distribution of SBMP

A particle size analysis of SBMP was performed using Mastersizer 3000 (Malvern Instruments Ltd., Malvern, UK) as per the method outlined by Mahadev and Meena [25]. This analysis provided values for specific surface area (SSA), particle size distribution (d_10_, d_50_ and d_90_), De Broukere (D_4,3_) and Sauter (D_3,2_) means; in addition, the span or dispersion index (span index = (d_90_ − d_10_)/d_50_) was also calculated. The refractive index (1.334) and density (1.30 g cm^−3^) of SBMP were determined and subsequently used for its particle size analysis.

### 2.8. Analysis of Antioxidant Properties of SBMP

The ABTS activity of SBMP was estimated according to the method reported by Salami et al. [34], while its DPPH and FRAP were determined by adopting the method reported by Zhang et al. [35] and Benzie and Strain [36], respectively. Total phenolic component and flavonoids present in SBMP were estimated by adopting the method described by Sharma et al. [37].

### 2.9. Fatty Acids Profiling of SBMP

A gas chromatography (GC) unit (model: GC-FID 2010 plus, make: Shimadzu, Japan) was used to estimate the fatty acid profile of SBMP. Fatty Acid Methyl Ester (FAME) derivatization was carried out with 20 mg of extracted fat from SBMP sample as per the ISO 15884 [38] method.

(All methods from the Section 2.3, Section 2.4, Section 2.5, Section 2.6, Section 2.7, Section 2.8 and Section 2.9 are discussed in brief in Supplementary Materials Section S1.)

### 2.10. Amino Acids Profiling of SBMP 

SBMP powder was passed through a sieve (80 mesh size) to screen for a uniform size. A total of 30 mg of SBMP was weighed in three replicates and transferred into the PTFE tube of a microwave digestion system. Then, 8 mL of 6 N hydrochloric acid was transferred into a PTFE tube. A SBMP powder sample was dissolved in 0.5 mL phenol, 1.0 mL of Nor-leucine internal standard (1000 ppm) and 0.5 mL of 0.1 N hydrochloric acid. Then, it was subjected for digestion, using microwave at 850 W. The digestion was achieved in two steps: ramping up the temperature to 160 °C for 15 min and then cooling the digested samples for 15 min.

Before pre-column derivatization, digested samples were diluted to 100 mL with 0.1 N hydrochloric acid solution. For pre-column derivatization, digested samples were mixed with 100 µL of borate buffer and 1.0 mL of 9-Fluorenylmethy1 chloroformate (FMOC-Cl, 0.4% solution) and kept undisturbed for 2 min. A total of 4.0 mL of n-pentane was added to the solution and then vortexed for 45 s. The upper layer was discarded, and the lower layer was transferred to the HPLC 1.5 mL vial. Amino acid profiling was conducted using reverse-phase UHPLC (Thermofisher Scientific Dionex Ultimate 3000, Waltham, MA, USA) with a C18 column (Acclaim T* 120, 5 pm, 120 A, (4.6 × 250 mm)) and photo diode array detector (Ultimate 3000 Diode Array) in the UV range. Mobile phase A consisted of 1800 mL of buffer and 200 mL of organic phase, while mobile phase B consisted of 200 mL of buffer and 1800 mL of organic phase. Here, the buffer was tetra-methy-l-ammonium chloride and sodium acetate trihydrate with a pH of 0.5, and the organic phase was mixture of acetonitrile and methanol in the ratio of 49:1. The gradient program used for the quaternary RS pump compartment is shown in Supplementary Table S1. The flow rate of the eluant was 1.0 mL/min, with a run time of 75 min. The response of the monitor was monitored at 265 nm, and data were acquired and processed by Chromeleon (6.8 SR1 5b; Build 4981).

### 2.11. FTIR Spectra of SBMP

An FTIR analysis of SBMP was conducted by its direct contact into Diamond crystal cell Attenuated Total Reflectance crystal of Shimadzu IR Affinity-1. According to Patil et al. [39], absorption spectra of SBMP was recorded between 4000 and 400 cm^−1^ wavenumbers at a 4 cm^−1^ resolution and a 0.2 cm s^−1^ scan speed, as per Patil et al. [39] Background run was taken before placing SBMP to the diamond crystal, as reported by Upadhyay et al. [40].

### 2.12. XRD Spectra of SBMP

X-ray diffraction (XRD) spectra were recorded for SBMP, using an X-ray diffractometer (model: MiniFlex II, make: Rigaku, Japan) equipped with a graphite reflected beam monochromator and PC-Automatic Powder Diffraction software version (APD, 3.0). It was operated in reflection mode at 40 kV and 50 mA. SBMP was slightly pressed on aluminum trays, using a 10 mm wide spatula (sample layer, 15 mm × 20 mm × 1.5 mm), and exposed to CuKα radiation (l = 0.15418 nm) at diffraction angles (2θ) from 10 to 80° (step size, 0.02°; time per step, 2.5 s). The divergence slit for the primary beam was 1°, and the divergence and receiving slits for the diffracted beam were 1° and 0.2 mm, respectively. The peak was searched for in APD software to locate the peaks in XRD patterns by detecting the minima from the second derivative of the diffractogram.

### 2.13. Microstructure of SBMP

The microstructure of SBMP was examined by scanning electron microscopy (EVO 50, Carl ZEISS Special Edition, Cambridge, UK), as per Shilpashree et al. [31].

### 2.14. Statistical Analysis

The results obtained during the processing of SBM in this investigation were subjected to a one-way analysis of variance (ANOVA), with a 5% level of significance (α = 0.05). Means were compared using Tukey’s HSD as a post hoc test in the IBM SPSS program, version 25. Wherever applicable, a descriptive statistics analysis was also performed and reported for SBMP parameters.

## 3. Results and Discussion

### 3.1. Chemical Composition of Buttermilk Samples and Concentration of DSBM

The concentration of DSBM from 1× to 3.62× (72% VRR) in the RO process gradually decreased the flux (Figure 2) and resulted in a 15.07 Lm^−2^ h^−1^ mean flux value, which corresponded to the critical flux of CSBM, and this was the reason for terminating the RO process at 3.62×. The concentration polarization and fouling of the RO membrane could explain such a reduction in flux. It is a well-established fact that the permeate flux decreases with the increase in the concentration factor or percent VRR due to an overall increase in the TS and viscosity of the RO concentrate.

The chemical composition and other physical properties of SBM, DSBM and CSBM are shown in Table 1. The acidity and pH values of pooled SBM were the values reported by Padghan et al. [9] for a similar product. Defatting significantly (*p* < 0.05) decreased the TS, fat, L*, a*, b* and ƞ_Appa_ of SBM as compared to DSBM. The dewatering of DSBM in the RO process significantly (*p* < 0.05) enhanced all of its chemical constituents and physical properties in CSBM (Table 1). Contrary to that, after a 3.62× concentration of DSBM, a significant (*p* < 0.05) decrease was observed in the ζ-potential of CSBM which could be attributed to its lower pH. A significant (*p* < 0.05) decrease in the pH of CSBM over DSBM was attributed to the concentration of lactic acid (LA) by RO. Li et al. [41] also successfully concentrated the LA present in nanofiltration (NF) permeate by employing RO. Govindasamy-Lucey et al. [15] also used RO to dewater sweet cream buttermilk (SCBM) containing 8.2% initial TS and achieved 21% TS after its 2.56× concentration.

**Figure 3:**
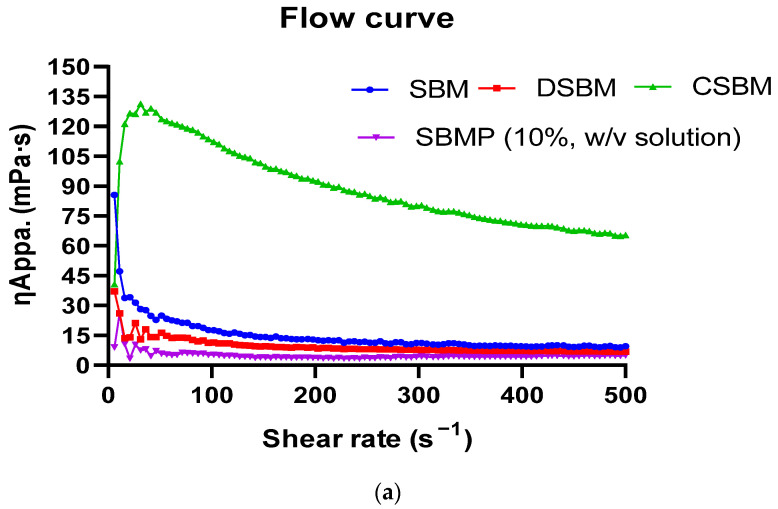

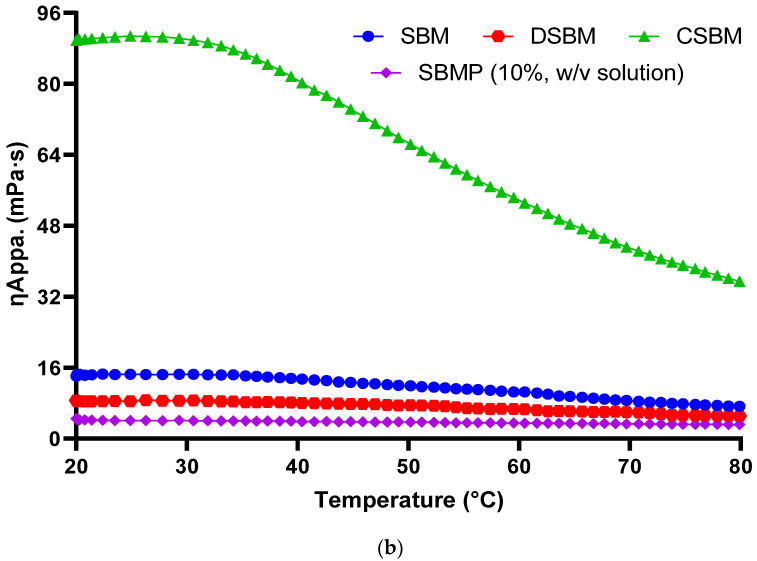
Change in viscosity of sour buttermilk (SBM), defatted sour buttermilk (DSBM), concentrated sour buttermilk (CSBM) and reconstituted (10% *w*/*v*) sour buttermilk powder (SBMP) samples as a function of increase in (**a**) shear rate and (**b**) temperature.

Defatting significantly (*p* < 0.05) decreased the TS, as well as the ƞ_Appa_, of DSBM as compared to SBM. This could have been attributed to the removal of milk fat and suspended milk solids. Opposite to this, the concentration of DSBM in RO significantly (*p* < 0.05) increased the TS and ƞ_Appa_ of CSBM. The ƞ_Appa_ of SBM, DSBM and CSBM exhibited a gradual decrease with the increasing shear rate and temperature, as demonstrated in Figure 3a,b, respectively.

### 3.2. Characterization of SBMP

#### 3.2.1. Chemical Composition and Physical Properties of SBMP

The production process of SBMP is outlined in Figure 1. Its chemical composition, physical, reconstitution, functional and antioxidative properties are shown in Table 2. Because of the lack of works in the scientific literature on powder manufactured from sour buttermilk (that too produced using traditional method), a the direct comparison of the results of the manufactured SBMP is not possible. Hence, its chemical composition and other powder properties were compared with related (lassi powder, yoghurt powder, buttermilk powder, skim milk powder (SMP) and whole milk powder (WMP)) products. Its TS (97.819%) content was in accordance with the TS content of *lassi* powder (94–97%; [42]), yoghurt powder (≥95%; [43]), SMP (97%; [44]), WMP (97.75%; [45]) and buttermilk powder (97%; [46]). SBMP had a markedly higher protein (53.64%, shown in Table 2) content over that which was reported for yogurt powder (35–38%; [43]), buttermilk powder (30–33%; [46]), SMP (34–37%; [44]) and WMP (24.5–33%; [45]). The fat content of SBMP was also higher than the fat contents (1.2–2.4%) of lassi (2.4%; [42]) and skim milk yoghurt powders (1–1.5%; [43]), respectively. Opposite to this, the lactose content of these powders (45–52% for yoghurt powder [43], 30–33% for buttermilk powder [46], 34–37% for SMP [44] and 24.5–27% for WMP [46]) was higher than that of SBMP (Table 2). Not much of the ash content of SBMP and yoghurt powders (6.8–8%; [43]) was comparable.

The reconstitution and flow properties of milk powders exhibit an inverse relationship with their free fat content. Powders also become prone to oxidation with an increase in their free fat content [47]. The lower fat content of CSBM was responsible for the lower free fat (1.23%) content of SBMP. The free fat content of WMP has been reported to be 2–3% [48] and <10% [49]. The Maillard reaction is a non-enzymatic browning reaction that occurs between milk sugar and proteins during the heating and drying of milk and even continues during the storage of milk powders. This also leads to a nutritional loss and change in color of milk and milk powders [50]. Hydroxymethylfurfural (HMF) compounds indicate the severity of the Maillard reaction [51]. The protein-to-lactose ratio and severity of the heat treatment greatly influence the formation of HMF in milk powder [52,53].

The higher HMF content of SBMP was mainly attributed to the prolonged heat treatment offered to milk from which SBM was produced. According to Sert et al. [54], the HMF content of SMP and WMP was 1.180 µmol/L and 0.715 µmol/L, respectively. The higher protein content, use of a higher processing temperature and low pH of the concentrate might have collectively contributed to a higher HMF content in SBMP. Stapelfeldt et al. [55] reported that the TBA value measures the lipid oxidation and determines the formation of secondary oxidation products (e.g., carbonyls). The formulated oxidative products further govern the sensory attributes of dairy powders. SBMP had a lower TBA value (Table 2) owing to the lower fat content of DSBM and CSBM (Table 1).

The acidity of the reconstituted SBMP solution (10%, *w*/*v*) was determined and expressed in terms of % LA (Table 2). The acidity of skim milk yoghurt powder and reconstituted lassi powder was 5–8% LA and 0.52–0.57%, LA, respectively [42,43]. The pH values of reconstituted SBMP, yoghurt powder and lassi powder solutions were 4.22 (Table 2), 4.3–5.3 [43] and 4.63–4.7 [42], respectively. Wade et al. [56] reported that the ζ-potential becomes less negative with the decrease in pH, and the same can easily explain lower the lower ζ-potential value of the SBMP solution, as shown in Table 2. The a_w_ of SBMP was 0.25, as shown in Table 2. According to Koc et al. [57], lower (<0.25) a_w_ values ensure powder stability during storage and also prevent microbial growth. For SMP and WMP, a_w_ values have been reported in the range of 0.1–0.303 and 0.23–0.32 by Szulc et al. [58] and Pugliese et al. [59].

The color of the powder plays an important role in its consumer acceptance and application. The color values of SBMP are shown in Table 2. The L*, a* and b* values of spray dried *dahi* powder, SMP and WMP were 93.6, 3.2 and 15.3 [60]; 96.94, −2.32 and 11.12; and 96.01, −1.74 and 14.45 [59]. As evident by its color values, SBMP was slightly brown in color, and this could be attributed to the formation of brownish pigments by Maillard reactions during the heat treatment of the milk [61] and spray drying of CSBM.

#### 3.2.2. Bulk and Flow Properties of SBMP

The volume difference between the mass of powder particles and volume of the same mass of tapped powder is known as the IAC, while the difference between the volume of the particles of the given mass and the volume of air-free solids is known as the OAC [62]. The IAC and OAC values of SBMP are shown in Table 2. As per Schuck [62], the IAC values of SMP and WMP were 41 and 120 cm^3^ 100 g^−1^ powder, respectively. The IAC of SBMP falls in between the IAC values of SMP and WMP. The degree of agglomeration and particle size distribution are the major factors which affect the IAC [62]. The OAC content of SBMP was 8.414 cm^3^ 100 g^−1^ powder (Table 2), which was markedly lower than the OAC content of SMP and WMP [62].

According to Schuck [62], the OAC content of SMP and WMP were 119 and 63 cm^3^ 100 g^−1^ powder, respectively. The OAC content of SBMP (Table 2) was markedly lower compared to these values. Indeed, 100 g of milk powder has been reported to contain 10–200 mL of OAC, which is influenced by feed properties (pH and protein content) and processing conditions such as the air incorporation/whipping of feed and its foam stability, as well as the type of atomizer used for its spray drying [62]. Zang and Goff [63] also reported that foaming decreases with the decrease in pH of the feed. Hence, severe heat treatment of milk prior to fermentation and the low pH (3.28) of CSBM could collectively explain the lower OAC content of SBMP.

The bulk characteristics of SBMP are shown in Table 2. The bulk characteristics (bulk and tapped densities, porosity and flowability) of a food powder rely heavily on particle size and its distribution [64]. A product with a low bulk density needs a larger packing volume. The LBD of skim milk yoghurt powder, skim milk yoghurt powder with a natural sour taste, normal yoghurt powder, SMP and WMP was 0.60–0.75 g/mL, 0.4–0.7 g/mL [43], 400 kg/m^3^ [65] and 431 and 360 kg m^−3^ [62], respectively.

The flowability is the ability of the powder to flow without forming any lump or aggregates [62]. The angle of repose (θ) is most commonly used to describe it. According to Carr [66], powders with θ values up to 35° have been classified as free-flowing. SBMP also exhibited flowability similar (free-flowing) to that of sand/salt. As per HR and CI values, SBMP demonstrated fair (CI, 16–20%; HR, 1.19–1.25) flow characteristics. For yoghurt powder, the reported CI value was 27.93 [65]. Schuck [62] reported that powder particles with larger particle diameters (>90 μm) demonstrate better flow characteristics. Hence, the presence of larger particles and lower fat content could explain the free-flow nature of SBMP.

The porosity of SBMP was 59.36% (Table 2), and the same was higher than the porosity (36.54%) of yoghurt powder [65], as well as that of WMP and SMP (52%) [47]. Fang et al. [67] reported that the presence of higher number of smaller particles causes a decrease in the porosity of the powder. Hence, the observed variation in particle size could explain the better porosity of SBMP.

#### 3.2.3. Reconstitution and Functional Properties of SBMP

The time taken by one gram of powder sample for the penetration of the still surface of water is known as wettability [62]. Factors such as particle density and size, powder porosity, surface area, surface charge and activity, as well as the presence of moisture-absorbing constituents, collectively influence the powder wettability. As per Kelly et al. [68], the wettability of WMP ranged between 30 and 60 s; meanwhile, SMP being wetted in <15 s deemed it to be an “instant powder”. The wettability of yoghurt powder developed by Koc et al. [65] was 374 s. The SBMP powder demonstrated wettability of only 3.00 s, which clearly indicated that it was an “instant powder”. As per Pimentel et al. [69], SBM is a good source of phospholipids (115.50 mg per 100 g); hence, the natural presence of surface-active agents (phospholipids particularly lecithin) could explain its excellent wettability.

Under standard testing conditions, the ability of powder particles to be uniformly dispersed in water is known as the dispersibility and confirms whether the powder is “instant” or not [62]. It is greatly influenced by heat-induced interactions between casein and whey proteins which may lead to the formation of an unstable dispersion [68]. As per Tammie [70], SMP possesses higher dispersibility (≥90%) compared to WMP (≥85%). Ji et al. [71] also reported that the dispersibility of SMP and WMP were 95 and 71%, respectively. SBMP had 73.74% dispersibility, as shown in Table 2. Koc et al. [65] reported that the dispersibility of spray-dried yoghurt powder was 351 s. Schokker et al. [72] reported that dried milk powders containing higher casein content were poorly dispersible and require a longer time for their complete dispersion. Singh and Newstead [73] reported that the dispersibility of milk powders is inversely proportional to the presence of fine particles (<90 µm). The heat-induced interactions between casein and whey proteins, higher HMF content and high protein content could collectively explain the intermediate dispersibility of SBMP (Table 2).

The solubility of milk powders can be expressed using different methods. Table 2 clearly shows that the solubility index of SBMP was 71.50%, which is higher than the reported solubility index value (68.70%) of yoghurt powder [65]. As per Schuck [30], a dairy powder is considered to be soluble if its solubility index is >99%. Lactose and salts are major hydrophilic constituents present in milk powders. The prolonged heat treatment of milk prior to fermentation and higher protein content of SBMP (Table 2) could be responsible for its relatively lower solubility index in comparison to SMP and WMP.

The interaction of proteins with water has been reported to determine the functional properties of proteins in different food systems. According to Zayas [74], the WBC of proteins is collectively influenced by several factors, namely the temperature, ionic strength, pH, concentration of proteins, lipids and salts, presence of hydrophilic polysaccharides, severity of heat treatment and storage conditions. Knightbridge and Goldman [75] reported that the method used for the drying and grinding of milk powders influences their topography, porosity and size and affects their water-holding capacity. The WBC of SBMP was 434 g water per 100 g protein, which was observed to be markedly higher compared to 33–180 g water per 100 g protein present in whey powders. The higher WBC of SBMP could be attributed to its higher protein content (Table 2) and prolonged heating of milk (advocating partial denaturation, dissociation and unfolding of protein) from which SBM and SBMP were produced. The severe heat treatment of whey proteins results in a higher WBC. Furthermore, Wagner and Anon [76] reported an inverse relationship between WHC and solubility. Indeed, denatured proteins result in a stable protein matrix in which a significant amount of water gets entrapped. A similar explanation holds true for the higher WBC of SBMP.

Similarly, the oil-binding capacity (OBC) is the ability to retain and absorb fat. It is influenced by the size of powder particles. Zayas [77] reported that protein powders possessing a low density and smaller particle size can adsorb and retain more oil over protein powders with a higher density. Hence, the higher LBD, PBD and PD of SBMP (Table 2) could explain its observed OBC.

Foaming capacity is the ability of protein solution to entrap the air bubbles or foam formation at the protein–water interface, whereas foam stability is the ability of milk protein to provide strength to foam lamella and retain the foam in the protein matrix [78]. The protein concentration, degree of denaturation, preheat treatment, ionic strength and lipid content collectively influence the foaming properties [79]. Both the foam capacity and foam stability of SBMP (Table 2) were poor and could be explained by its lower pH, lower ζ-potential and lower solubility index. This is because the foaming capacity of proteins is directly proportional to the net proton charge present on them.

The emulsifying capability (EC) refers to the ability of protein solutions to emulsify at the oil–water interface via adsorbing the oil molecules at their surface [80]. The emulsion stability (ES) is the ability of emulsion droplets to remain distributed without aggregating, flocculating or creaming [81]. Emulsification properties are influenced by several factors, including TS, pH, protein, calcium content and powder particle size [82]. The ability of a protein to act as an emulsifier majorly depends on its amphipathic nature, solubility, extent of surface denaturation and lipid-to-protein ratio [83]. Several factors, such as the presence of MFGM and phospholipids, which are known as natural emulsifiers, as well as lower solubility of SBMP, could be responsible for higher EC and ES (Table 2) values.

The apparent viscosity of reconstituted SBMP solution was measured and is shown in Table 2. In the case of yoghurt powder, the consistency of reconstituted yoghurt solution was thinner than that of original yoghurt [84]. This may be due to extensive heat treatment during spray drying which results in the denaturation of milk protein. This showed poor rheological attributes during rehydration [85]. Hence, similar changes could also explain the three-times-lower apparent viscosity of SBMP (Table 2) compared to SBM (Table 1).

**Figure 4:**
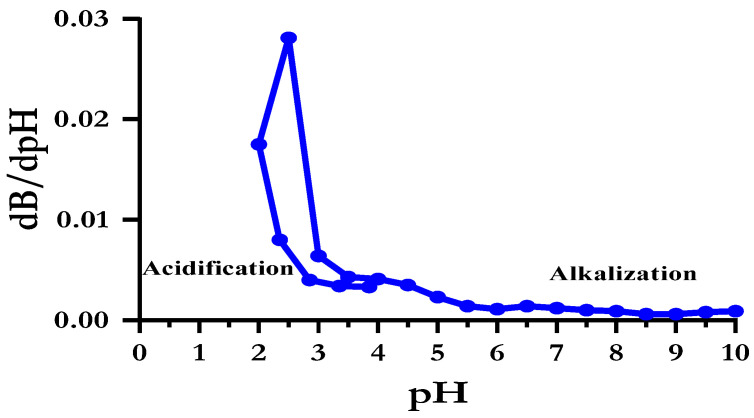
Buffering capacity of reconstituted sour buttermilk powder (SBMP) solution.

The buffering capacity is the typical property of milk proteins to resist a change in pH value. The buffering capacity of the SBMP solution (0.5% protein) is shown in Figure 4. Salaün et al. [86] reported that casein, whey proteins, colloidal calcium phosphate and soluble minerals contribute 35%, 5%, 20% and 40% of the buffering capacity in milk powders. Hence, its buffering capacity can be explained by its chemical composition, as shown in Table 2.

#### 3.2.4. Particle Size Distribution of SBMP

The appearance, reconstitution properties, flow characteristics and surface reactivity of milk powder are collectively explained by its particle size distribution. The processing conditions, original feed characteristics and use of different equipment during processing including drying are the key factors that influence particle size distribution [62].

**Figure 5:**
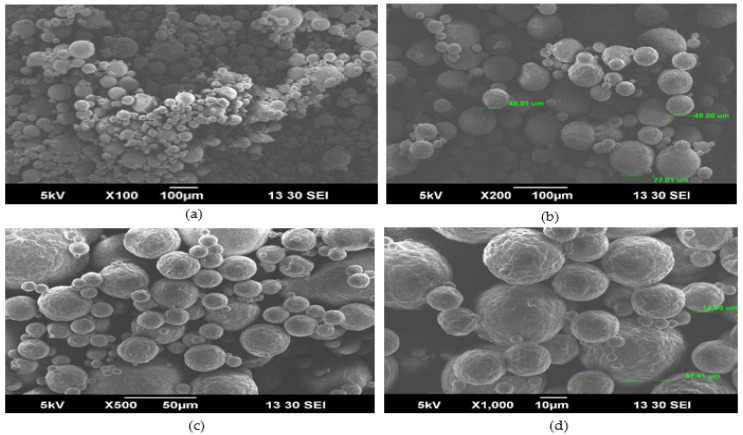
SEM images of sour buttermilk powder (SBMP) at (**a**) 100×, (**b**) 200×, (**c**) 500× and (**d**) 1000×.

Table 2 clearly represents the particle size distribution of SBMP. A higher number of particles (d_10_, d_50_ and d_90_) with a lower specific surface area (SSA) and span were observed for SBMP. The d_10_ of SBMP (Table 2) was higher than that of (27.15 µm) SMP but lower than that of (86.10 µm) WMP [59]. Furthermore, its d_50_ was lower than that of both SMP (83.87 µm) and WMP (128.76 µm), as reported by Pugliese et al. [59]. Similar findings were also observed for its d_90_, as shown in Table 2. The d_90_ values of SMP and WMP were 191.55 µm and 287.40 µm, respectively [59]. The D_4,3_ value of SBMP (Table 2) was found to be lower than that of SMP (9.56 µm) and WMP (49.17 µm), whereas the D_3,2_ value was higher than that of SMP (151.33 µm) but found to be lower than that of WMP (86.10 µm) [59], as reported in Table 2. The observed variation in the particle size distribution of SBMP could be attributed to the low total solids and viscosity of feed. The SEM micrograph (Figure 5) of the manufactured SBMP also indicates a porous structure and variability in their particle size, with a significant number of smaller-sized particles.

#### 3.2.5. Antioxidant Properties of SBMP

The ABTS value of SBMP (Table 2) was lower compared to the reported 57% [87] and 75% RSA [88] values for SMP. Meanwhile, the DPPH value for SBMP (Table 2) was higher than that of (77–94%) WMP [89], (3.26%) buffalo milk powder [90] and (2.87%) cow milk powder [90]. Zivkovic et al. [91] reported that the lipid content, polyphenol level, casein and whey protein contribute to the radical scavenging activity of milk. Padhgan et al. [9] reported that prolonged heat treatment before the fermentation of milk leads to the formation of a brown pigment and also liberates -SH groups. According to Taylor and Richardson [92] and Tong et al. [93], the protein’s unfolding and exposure to thiol groups may function as hydrogen donors during thermal treatments, which may result in an increase in antioxidant activity. Sarmadi and Ismail [94] reported that hydrophobic amino acids, including aromatic amino acids, can increase the radical scavenging activity. The FRAP activity of manufactured SBMP (Table 2) was higher than the FRAP values reported by Bhardwaj et al. [95] for Poitu (French breed) donkey milk powder (101.95 μmole/L) and that of Halari (Indian breed) donkey milk powder (74.62 μmole/L). Milincic et al. [96] reported that SMP produced from goat milk and enriched with grape pomace showed the ability to reduce the ferric ions due to the action of free phenolic compounds (such as gallic acids). Hence, the presence of phenolic compounds (Table 2) in SBMP could be responsible for its ferric-reducing antioxidant power.

The total phenolic compound content of SBMP (Table 2) was higher than that of WMP (8.1–9.8 mg/L) [89], SMP (163.75–96.48 μM GE/L) [87], cow milk powder (0.49 mg GAE/g) and buffalo milk powder (3.26 mg GAE/g) [90]. The presence of phenolic compounds at high levels in cattle feed and the microbial activity in milk are liable for the presence of phenolic compounds in milk [97]. This explanation could be true for the observed total phenolic content of SBMP. Additionally, the flavonoid content of SBMP (Table 2) was found to be slightly higher than that of cow milk powder (0.05 mg RE/g) and (0.04 mg RE/g) buffalo milk powder [90]. It is reported that dietary supplementation of dairy feeds with *Scutekkaria baicaolensis* [98], soybean oil or grapes pomace [99], or feeding of fruit [100], could lead to a change in the antioxidant profile in milk and milk products. 

#### 3.2.6. Fatty Acid Composition of SBMP

Table 3 and Supplementary Figure S1 reveal the presence of short-, medium- and long-chain fatty acids in SBMP. It contained a major proportion of palmitic acid (46.89%), myristic acid (15.88%) and stearic acid (13.47%). Halder et al. [6] reported that the use of a high temperature during the churning of curd via the traditional method yields a higher proportion of high-melting-point fatty acids over low-melting-point fatty acids. This could explain the observed difference in concentrations of fatty acids in SBMP. Marconi and Panfili [101] also observed a maximum concentration of palmitic acid (33.8%), followed by oleic acid (25.4%) and steric acid (12.7%), in cow milk powder. It is evident in our results also that the saturated fatty acids with a higher melting point that consisted of palmitic, myristic and stearic were markedly higher than the unsaturated fatty acids, such as oleic acid (0.434%), linoleic acid (1.32%) and linolenic acid (0.47%).

#### 3.2.7. Amino Acid Composition of SBMP

The amino acid profiling of SBMP is shown in Table 4 and Supplementary Figure S2, which reveals the presence of different amino acids in different concentrations. Essential amino acids (histidine and threonine) were present in higher concentrations in SBMP than the values reported for SMP (0.92%, 1.61%) and WMP (0.66%, 1.16%) [102]. Germini et al. [103] reported that proteins partially hydrolyze into peptides and free amino acids during fermentation. Additionally, it is reported that free amino acids are produced during the first four hours of fermentation. After that, there is a decline of amino acid content because the bacterial use during the yoghurt’s production generates aromatic substances. Acetaldehyde is the primary component of the aroma produced by *L. bulgaricus*, whereas *S. thermophilus* mostly produces diketones in yoghurt. The uncontrolled fermentation of milk during curd making released free amino acids, and many of them were utilized by the lactic acid bacteria, as reported by Padgyan et al. [9].

#### 3.2.8. Fourier-Transform Infrared Spectroscopy (FTIR) Spectra of SBMP

The fundamental concept behind infrared spectroscopy is that all molecules absorb frequencies due to their molecular makeup [104]. In order to characterize the organic components in a food system (solid, liquid, or gas), FTIR is a very helpful approach [105].

**Figure 6:**
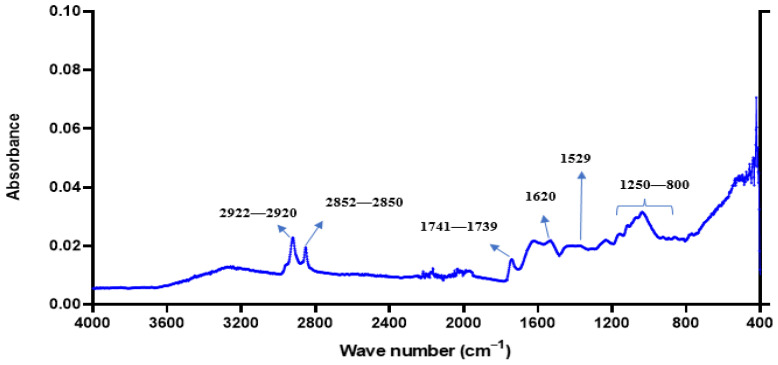
FTIR absorbance spectra of sour buttermilk powder (SBMP).

The primary constituents, fat, protein and lactose, each have distinctive peaks. IR peaks at 2920–2922, 2850–2852 and 1739–1741 cm^−1^ (Figure 6) emerge from the SBMP’s fat. As the fat concentrations were reduced, the C=O bond’s distinctive peak at 1739–1741 cm^−1^ intensified, and the vibrational frequency of C-O in fat, 1161 cm^−1^, noticeably drops. Therefore, a lower concentration of fat in the developed SBMP was also revealed by the spectra. The protein’s amide I and II vibrations of SBMP are represented by two broad peaks with middle intensities of 1620 and 1529 cm^−1^ (Figure 6), respectively, suggesting the higher concentration of milk protein in SBMP. The characteristic peaks of various C-O vibrations in carbohydrates are located between 800 and 1250 cm^−1^ (Figure 6), indicating the presence of milk lactose [106].

#### 3.2.9. XRD Spectra of SBMP

X-ray diffraction (XRD) is a frequently used rapid analytical technique for identifying the phase of crystalline materials [107]. It can also detect all forms of lactose crystallization. The manufactured SBMP powder was analyzed to determine the crystallization pattern of its amorphous constituents. Jouppila et al. [108] reported that amorphous lactose crystallizes into different forms, such as α-lactose monohydrate, anhydrous β-lactose, anhydrous α-lactose and an anhydrous mixture of α and β-lactose in a molar ratio of 5:3, 3:2 and 4:1, respectively. According to Nijdam et al. [109], the amorphous lactose in dairy powders crystallized as α-lactose monohydrate and a 5:3 molar combination of α- lactose and β-lactose.

**Figure 7:**
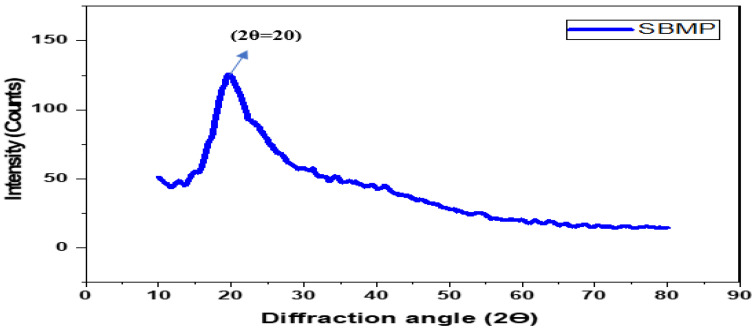
XRD spectra of sour buttermilk powder (SBMP).

The presence of lactose crystals in SBMP was identified using the location (denoted by diffraction angle, 2θ) and intensity data of the peaks observed in XRD patterns, as depicted in Figure 7. The results were compared with the literature related to different crystalline forms of lactose in skim milk. The available scientific literature on XRD patterns showed peaks of α-lactose monohydrate, stable anhydrous α-lactose, and anhydrous α and β-lactose mixture (molar ratio of 5:3) at a diffraction angle of 19.1° [110], 20.0° [111] and 20.1° [112], respectively. The lactose crystallization peaks of the XRD pattern for SBMP were observed (Figure 7) at a 20° diffraction, with a subsequent intensity value of 156.67. Hence, 2θ value of SBMP sample directly advocating the presence of crystalline lactose in the sample, which eventually falls in the range (19.1° to 20.1°) reported in the scientific literature.

#### 3.2.10. Particle Morphology of SBMP

SEM micrographs of SBMP (Figure 5) showed the presence of spherical-shaped powder particles of variable size. The particle size distribution (Table 2) also advocated the variation in the size of SBMP particles. Moreover, it showed some particle infusion and clustering that could be the cause of poor dispersibility and solubility. Furthermore, powder particles were attached in a clustered manner that could be attributed to amorphous lactose and surface fat. Hence, the majority of smaller particles also has a lower bulk density and higher IAC values (Table 2). This could be attributed to the lower TS content of the CSBM (Table 1) that was spray-dried to obtain SBMP.

## 4. Conclusions

SBM is criticized for its low TS, high acidity and poor thermal stability. Its concentration by RO markedly enhanced total milk solids in retentate, which were converted into stable SBMP via spray drying. SBMP was an “instant powder” in wettability, and it exhibits excellent flowability (θ), good porosity, immediate dispersibility, lower solubility index, lower foam stability and capacity, higher emulsification capacity and emulsification stability. Additionally, it contains essential amino acids and fatty acids and shows good antioxidant properties. SBM, DSBM, CSBM (RO concentrate) and SBMP solution exhibited typical shear-thinning behavior, and their apparent viscosity decreased with the rise in temperature. SBMP particles were smooth, variable in size and exhibited slight infusion and clustering. Overall, this study established that RO combined with spray drying can be used to valorize SBM into a novel dairy ingredient demonstrating excellent wettability, flowability, emulsification stability and antioxidant properties. The manufactured powder could be used to improve nutritional properties, viscosity, foaming capacity and stability of processed foods. However, its dispersibility, solubility index, oil-binding capacity and foam stability must be improved to further enhance its uses in different food formulations.

## Figures and Tables

**Table 1:** Chemical composition and physical properties of SBM, DSBM and CSBM samples.

Parameters		Sour Buttermilk (SBM)	Defatted Sour Buttermilk (DSBM)	Concentrated Sour Buttermilk (CSBM)
TS (%)		4.13 ± 0.14 ^b^	3.55 ± 0.12 ^c^	12.86 ± 0.31 ^a^
Fat (%)		0.72 ± 0.29 ^a^	0.18 ± 0.17 ^b^	0.67 ± 0.28 ^a^
Protein (%)		1.88 ± 0.19 ^b^	1.91 ± 0.10 ^b^	6.93 ± 0.39 ^a^
Lactose (%)		1.09 ± 0.33 ^b^	1.15 ± 0.24 ^b^	4.08 ± 0.30 ^a^
Ash (%)		0.23 ± 0.12 ^b^	0.28 ± 0.13 ^b^	0.98 ± 0.09 ^a^
Acidity (% LA)		0.70 ± 0.08 ^b^	0.70 ± 0.06 ^b^	2.44 ± 0.15 ^a^
pH, 20 °C		3.95 ± 0.07 ^a^	3.95 ± 0.07 ^a^	3.28 ± 0.09 ^b^
ζ-potential (mV) (100× dilution), 25 °C		11.20 ± 6.15 ^a^	13.85 ± 1.14 ^a^	8.88 ± 2.14 ^b^
Color values	L*	76.38 ± 0.20 ^c^	76.93 ± 0.22 ^b^	80.39 ± 0.44 ^a^
	a*	−1.96 ± 0.04 ^b^	−2.18 ± 0.15 ^c^	0.12 ± 0.85 ^a^
	b*	8.37 ± 0.50 ^b^	8.19 ± 0.45 ^c^	14.12 ± 0.06 ^a^
Apparent viscosity (ƞ_Appa._), 100 s^−1^ (mPa s), at 20 °C		14.10 ± 1.22 ^b^	8.47 ± 0.32 ^c^	90.66 ± 2.08 ^a^

Mean ± SD (*n* = 3) with different superscripts; ^a–c^ are significantly different (*p* < 0.05) from each other column-wise.

**Table 2:** Physicochemical, reconstitution, functional and antioxidative properties of SBMP (Mean ± S.D.; *n* = 3 independent trails).

	Parameters		SBMP
Chemical composition and physical properties	TS (%)		97.82 ± 0.18
	Fat (%)		5.04 ± 0.23
	Protein (%)		53.64 ± 0.31
	Lactose (%)		31.34 ± 0.60
	Ash (%)		7.48 ± 0.12
	Free fat (% of total fat)		1.23 ± 0.13
	Hydroxymethylfurfural (µmol/kg of powder)		1085.17 ± 1.61
	2-thiobarbituric acid, TBA (µg/mL)		0.12 ± 0.03
	Acidity (% LA)		1.80 ± 0.12
	pH (10% *w*/*v* solution), at 20 °C		4.22 ± 0.10
	ζ-potential (mV) (1000× dilution)		−0.18 ± 0.17
	Water activity (a_w_)		0.25 ± 0.04
	Color values	L*	74.69 ± 0.27
		a*	1.65 ± 0.15
		b*	26.62 ± 0.14
Bulk and flow properties	Interstitial air content (cm^3^ 100 g^−1^ powder)		74.62 ± 3.68
	Occluded air content (cm^3^ 100 g^−1^ powder)		8.41 ± 0.57
	Loose bulk density (g cm^−3^)		0.53 ± 0.10
	Packed bulk density (g cm^−3^)		0.66 ± 0.14
	Particle density (g cm^−3^)		1.30 ± 0.16
	Porosity (%)		59.36 ± 0.31
	Flowability (angle of repose, θ°)		28.36 ± 0.48
	Hausner ratio (HR)		1.23 ± 0.01
	Compressibility index (CI)		18.83 ± 0.60
Reconstitution and functional properties	Wettability (s)		03.00 ± 0.00
	Dispersibility (%)		73.74 ± 0.70
	Solubility index (mL per 100 mL reconstituted)		71.50 ± 0.20
	Water binding capacity (g per g of protein)		4.34 ± 0.63
	Oil binding capacity (g per g of protein)		2.77 ± 0.46
	Foaming capacity (%)		22.18 ± 2.82
	Foam stability (%)		14.32 ± 0.90
	Emulsion capacity (%)		32.05 ± 0.24
	Emulsion stability (%)		80.70 ± 0.79
	ƞ_Appa._(mPa s), at 100 s^−1^ and 20 °C		4.09 ± 0.74
Particle size distribution	d_10_		33.43 ± 0.11
	d_50_		69.17 ± 0.32
	d_90_		130.33 ± 2.89
	D_3,2_		91.23 ± 3.38
	D_4,3_		4.26 ± 0.01
	Span (%, dispersion index)		1.06 ± 0.01
	SSA (m^2^ kg^−1^)		65.83 ± 2.47
Antioxidant properties	ABTS (% RSA)		50.65 ± 0.47
	DPPH (%)		120.19 ± 0.42
	FRAP (µM/mL)		110.13 ± 0.64
	Total phenolic content (µg/mL)		1699 ± 6.08
	Flavonoids (µg/mL)		0.85 ± 0.12

**Table 3:** Fatty acid profiling of sour buttermilk powder (SBMP).

Types	Fatty Acids	% Total Fatty Acids
Short-chain fatty acids	Butyric acid (C4:0)	4.58
	Caproic acid (C6:0)	3.28
	Caprylic acid (C8:0)	1.42
	Capric acid (C10:0)	2.90
Medium-chain fatty acids	Lauric acid (C12:0)	3.57
	Myristic acid (C14:0)	15.88
	Myristoleic acid (C14:1) ^#^	0.87
Long-chain fatty acids	Pentadecylic acid (C15:0) *	1.45
	Palmitic acid (C16:0) *	46.89
	Hexadecenoic acid (C16:1)	2.33
	Margaric acid (C17:0) *	0.68
	Stearic acid (C18:0) *	13.47
	Oleic acid (C18:1) ^#^	0.434
	Linoleic acid (C18:2) ^#^	1.32
	Linolenic acid(C18:3) ^#^	0.47
	Arachidic acid (C20:0) *	0.46

* Saturated fatty acids; ^#^ unsaturated fatty acids.

**Table 4:** Amino acid profiling of sour buttermilk powder (SBMP).

Name	Peak Area mAU * min	Area Ratio (Std/IS)	Mg per g of Powder	mg per g of Protein
Argenine	2.2171	0.122	2.12	3.95
Serine	0.0127	0.274	2.68	4.99
Aspartic acid	0.008	0.361	5.41	10.08
Glutamic acid	3.4555	0.190	2.97	5.54
Threonine *	0.0002	0.153	2.07	3.85
Glycine	3.0864	0.169	1.24	2.31
Alanine	51.474	2.824	27.17	50.65
Proline	0.733	0.040	0.45	0.84
Methionine *	0.4738	0.026	0.40	0.75
Valine *	3.1443	0.172	1.97	3.68
Phenylalanine *	118.2526	6.487	120.18	224.04
Iso-Leucine *	30.1234	1.652	23.74	44.26
Leucine *	2.0047	0.110	1.50	2.80
Cystine	1.9833	0.109	16.04	29.91
Histidine *	5.677	0.311	2.62	4.88
Lysine *	0.3186	0.017	0.13	0.24
Nor-Leucine (IS)	18.23	1.000	131.74	245.58

* Essential amino acids.

## Data Availability

Data will be provided upon request.

## Supplementary Materials

The following supporting information can be downloaded at https://www.mdpi.com/article/10.3390/membranes13090799/s1, Figure S1: Fatty acids profiling chromagraph of Sour buttermilk powder (SBMP); Figure S2: Amino acids profiling chromagraph of Sour buttermilk powder (SBMP);Table S1: Gradient program used for Quaternary RS Pump Compartment; Section S1: Methods.

## Abbreviations

RO	reverse osmosis
SBM	sour buttermilk
TS	total solids
SBMP	sour buttermilk powder
SCBM	sweet cream buttermilk
DSBM	defatted sour buttermilk
CSBM	sour buttermilk concentrate
VRR	volume reduction ratio
CF	concentration factor
FM	flux mean
IF	initial flux
FF	final flux
IAC	interstitial air content
OAC	occluded air content
LBD	loose bulk density
PBD	packed or tapped bulk density
HR	Hausner ratio
CI	Carr index
SI	solubility index
WBC	water binding capacity
OBC	oil binding capacity
ƞAppa	apparent viscosity
SSA	specific surface area
XRD	X-ray diffractometer
SMP	skim milk powder
WMP	whole milk powder
HMF	hydroxymethylfurfural
TBA	2-thiobarbituric acid
EC	emulsifying capability
ES	emulsion stability
