# Supplementary material for: Valorization of Sour Buttermilk (A Potential Waste Stream): Conversion to Powder Employing Reverse Osmosis and Spray Drying
Source: Membranes (Basel). 2023 Sep 17;13(9):799. doi: 10.3390/membranes13090799 (PMC10534478; doi:10.3390/membranes13090799)
Supplement: Supplementary file 1 [file membranes-13-00799-s001.zip › membranes-2566434-supplementary.pdf]

Supplementary material

Valorisation of sour buttermilk (a potential waste-stream): conversion to powder employing reverse osmosis and spray drying

Subhadip Manik [1], Ganga Sahay Meena [1]*, Ashish Kumar Singh [1], Yogesh Khetra [1], Richa Singh [2], Sumit Arora [2] and Raghu H.V [3]

[1]Dairy Technology Division, ICAR-National Dairy Research Institute, Karnal, Haryana, India
[2]Dairy Chemistry Division, ICAR-National Dairy Research Institute, Karnal, Haryana, India
[3]Dairy Microbiology Division ICAR-National Dairy Research Institute, Karnal, Haryana, India
* Correspondence: Ganga Sahay Meena; Email: : ganga.meena@icar.gov.in; Contact No: +91- 9996129094.

Table S1. Gradient program used for Quaternary RS Pump Compartment.

Time (in Min)	%A	%B
0	90	10
5	76	24
10	76	24
45	50	50
50	50	50
60	10	90
62	0	100
67	0	100
69	90	10
75	90	10

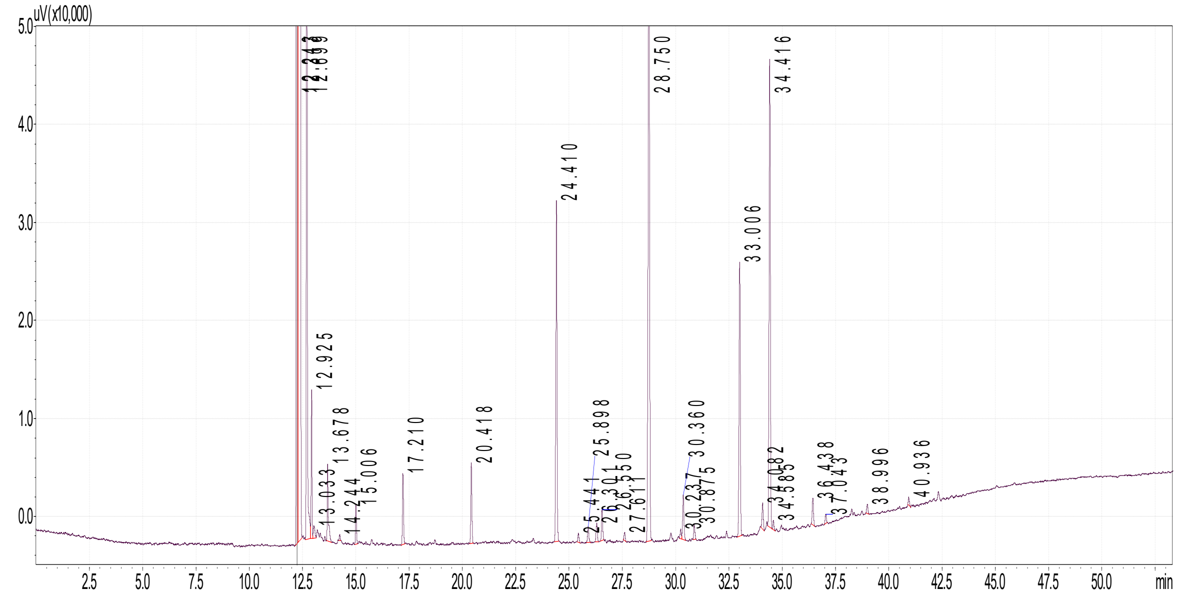

Figure S1. Fatty acids profiling chromagraph of Sour buttermilk powder (SBMP).

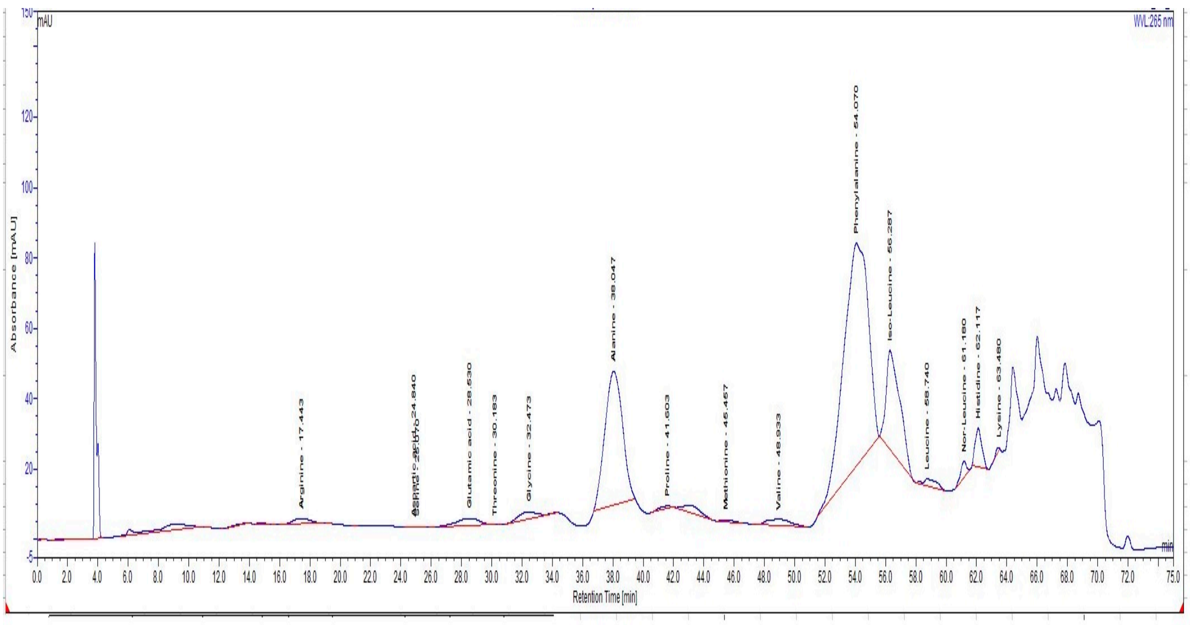

Figure S2. Amino acids profiling chromagraph of Sour buttermilk powder (SBMP).

Section S1: Methods

ζ-potential

ζ-potential was estimated as per the method described by Mahadev and Meena [25]. Here, samples such as SBM, DSBM, CSBM were diluted with Milli-Q water(1:100) while SBMP samples were diluted with Milli-Q water (1:1000) and subjected for vortexed (10-15 s) and introduced to the Zetasizer Nano ZS, (Malvern WR14 1XZ, UK) at 25 °C.

Free fat content of SBMP samples

To determine the free fat content of SBMP samples, Hall and Hedrick[21] suggested a method with slight modification, i.e., instead of 2.5 g powder, 10 g powder was used. The powder was taken in a 250 mL conical flask, and 50 mL of petroleum ether was added. The solvent was separated by filtration into a round-bottom flask through a Whatman filter paper grade no 40. The filtrates were dried and kept in a hot air oven for encapsulated fat analysis. First, the solvent in the flask was evaporated. Then, the solvent-free content inside the flask was dried in the oven. Finally, the free fat percentage was calculated by following the formula:

$$Free\ fat\ (\%) = \frac{(W3 - W2)}{W1} \times 100$$

Where,

W1= Weight in g for powder sample

W2= Weight in g for empty flask

W3= weight g for empty flask + extracted matter

Hydroxymethylfurfural content

Hydroxymethylfurfural (HMF) of SKP was estimated by Method B of Keeney & Bassette [21]. First, 1g powder samples were reconstituted with 10 mL water and were then transferred to test tube (volume 50 mL), followed by the addition of 5 mL freshly prepared 0.3N oxalic acid and kept in a boiling water bath for 60 min. The tubes were then cooled to room temperature, and 5 mL of trichloroacetic acid (40% of strength) solution was added. The content was mixed vigorously and filtered using Whatman filter paper (No. 42). Four mL of the filtrate was transferred to a 10 mL stoppered glass tube, and 1 mL of 0.05 M aqueous solution of TBA was then added, the tubes were stoppered and their contents mixed thoroughly before keeping them in a water-bath at 40±1oC for 40 min. The tubes were then cooled to room temperature, and absorbance was measured at 443 nm in a 10 mm cell using the UV-VIS spectrophotometer. Next, a blank was carried out, taking distilled water instead of a sample. The standard HMF was procured from Hi-Media

Laboratories Pvt. Ltd, Mumbai, and a standard curve of HMF concentration (absorbance at 443 nm) was prepared, and a reading was taken by UV-VIS spectrophotometer. A standard stock solution of 2500 μM HMF was made into distilled water, and dilution ranging from 500-2250 μM was made using distilled water. The samples were covered with aluminium foil to protect them from lights induced oxidation. HMF concentration was determined using the equation derived from the standard curve.

2-thiobarbituric acid (TBA) value

2-thiobarbituric acid were measured using the method described by Hegenauer et al. [23] with slight modification, i.e., 1.0 g of SBMP was used here. Freshly prepared TBA 0.025 M TBA (neutralized with NaOH) solution and 2 M citric acid solution in 1:1 ratio. 1g SBMP powder was mixed thoroughly with 2.5 mL of TBA reagent and subject for heat treatment in boiling water bath for 10 min followed by cooling in ice bath. Thereafter, 10 mL of cyclohexanone and 1mL of 4M ammonium sulfate were added and centrifuged at 8200 rpm for 5 min at room temperature (30 °C). The orange-red cyclohexanone supernatant was decanted and its absorbance was recorded at 532 nm in a spectrophotometer and the TBA value was expressed as μg per mL and a standard curve was prepared by using 0.1to 3ug/mL MDA solution.

Color value

Before the test, the instrument was calibrated with standard black and white tiles as specified. Measurements were then made on the SBM, DSBM, CSBM and SBMP samples taken in a glass sample cup supplied with the instrument by filling it to a fixed level for each sample. Data were received through the software in terms of L*, a*, and b* (yellowness) values. During color measurement, special care was taken to avoid breaking the sampling cup. Color values were taken in triplicate per sample was noted down.

Water activity (a_w)

a_w of SBMP was measured using a water activity meter. Firstly, the instrument was calibrated by placing the dry charcoal into the sample tray to absorb the internal moisture of the chamber. Next, the sample was quickly transferred into the sample tray to prevent moisture gain. Finally, the tray was placed in the instrument, and the chamber was sealed for measurement. The readings were taken in triplicates.

Interstitial air content (IAC) and Occluded air content (OAC)

The IAC and OAC of SBMP samples were determined by adopting the method reported by Niro Atomizer [26]. The IAC and OAC were calculated using the following formula:

$$V\,ia = \frac{100}{D\,packed} - \frac{100}{D\,particle}$$

Where,

V_{ia} = volume of interstitial air in 100 g of powder, cm3

D powder = packed bulk density of powder solids, g/cm3

D particle = mean particle density, g/cm3

$$V\,oa = \frac{100}{Dpowder} - \frac{100}{D\,particle}$$

Where,

Voa = Volume of occluded air in 100 g of powder, cm3

D particle = Mean particle density, g/cm3

D solid = Density of powder solids, g/cm3

$$D\,solids = \frac{100}{\%\frac{Fat}{0.93} + \%\frac{SNF}{1.52} + \%\frac{Water}{1}}$$

Particle density (PD)

PD of SBMP was determined by adopting Niro Atomizer [26]. In this method, 25 g of SBMP was taken into a 100 mL measuring cylinder containing a glass stopper. Thereafter, 50 mL of petroleum ether (BP 40-60 °C) was added and shaken until all the powder particles were dispersed. Powder particles present into the wall of measuring cylinder were

scraped down using a rubber spatula. Rinsing of spatula and walls was accomplished with the addition of 10 mL of petroleum ether using a pipette making the total volume of ether 60 mL. The total volume of petroleum ether and suspended powder particles was recorded. Particle density in g.cm3 was calculated as per the following formula:

$$D\ particles = \frac{W}{V - 60}$$

LBD and PBD

LBD and PBD of SBMP sample were calculated using the described procedure by Sjollema [27]. First, tare weight of a 100 mL graduated cylinder was taken. Then, a funnel was placed over a cylinder opening, and SBMP was allowed to flow freely through the funnel up to the 100 mL mark. Next, cylinder containing with powder was recorded. The net weight of 100 mL powder was expressed as LBD (g. cm-3). Furthermore, it was tapped 100 times using the tap density tester, and the resultant volume was recorded and expressed as PBD (g. cm-^{3}).

Porosity

The porosity of SBMP samples was calculated using their LBD and PD as reported by Sjollema [27] as per following formula:

$$Porosity\ (\%) = \frac{100x(PD - LBD)}{PD}$$

Flowability

The angle of repose of SBMP was determined using method of Sjollema [27]. A plastic funnel with narrow stem and cut at right angles, mounted on a butter paper at exactly 2 cm above as well as positioned on a horizontal table. SBMP was allowed to fall through the funnel in a fine stream at a controlled speed, so a conical heap was formed beneath the funnel. The powder addition was stopped when top of the powder heap touched the end of the funnel stem. Then, powder base was outlined with pencil and it was recovered. The diameter of the portion marked with a pencil was recorded at different points to calculate its radius, and its angle of repose was calculated using the following formula:

$$tan\ \theta = \frac{h}{r}$$

$$\theta = tan - 1(\frac{h}{r})$$

Where,

Θ = Angle of repose

h = Height of stem base (Fixed at 2cm) r = Radius of the powder heap (cm)

Wettability

The wettability of the SBMP samples were measured using the method by Muers & House [28]. A piece of the fabric (10 cm × 10 cm) was stretched over one end of the body of a metallic can (6.5 diameter and 4.5 cm height), open at both ends and held on with a rubber band. Another open end can (5cm diameter and 7 cm height) was placed as a spacer to hold it centrally on the cloth. The tray (21.2 of length ×16.3 cm of width) was marked at a depth of 2.5cm from the bottom and filled with distilled water at 40 °C. A triangular set up of 0.4 cm thick glass rod with a side stand was placed in the dish, which served to prevent close contact of cloth with the bottom of the dish. With two cans assembled and the cloth resting on a clean surface, 1 g of SBMP was transferred to the inner can and spread over the 5 cm circle of cloth as evenly as possible with a soft hair brush. The inner can was then removed, and the outer can was lowered into the dish onto the glass triangle and held in place until the water level in the can ceased to rise. A stopwatch was started when the cloth touched the water and was stopped when the powder was completely wetted. The mean of 3 replicates was taken as the wetting time and reported in seconds.

Dispersibility

Dispersibility of SBMP was measured using the method of ADMI [29]. A brass bowl (height of 13cm, 13cm of top diameter and 24 cm of inner diameter) fitted with an outlet of 1.25 cm diameter in bottom assembled with a rubber tube with pinch-cock was taken. This was clamped to a stand for its raising or lowering. A total of 400 mL of water (40 °C) was placed into the bowl, and 56 g of SBMP sample was transferred to the surface of the

water. A motorized stirrer (Remi Udyog, Mumbai, India) of the mixer whose blades were earlier fitted in position in the centre of the bowl was operated at 400 rpm for 90s. The pinch cock of the bowl bottom outlet was opened to release mixed sample and passed through a standard sieve (72 mesh). It was collected into a 500 mL volumetric flask and diluted up to 500 mL. TS was measured via duplicate 10 mL of this portions. Total solids weight was multiplied with 50 to obtain dispersibility in grams. Per cent dispersibility was calculated using the following formula:

$$Dispersibility\ (\%) = \frac{Dispersibility\ in\ g}{56} \times 100$$

Water binding capacity (WBC) and Oil binding capacity (OBC)

The WBC and OBC of SBMP was determined using the method described by Shilpashree et al. [31]. WBC and OBC was determined by mixing 3 g of protein SBMP with 10 mL deionized water and refined soybean oil in a pre-weighed centrifuge tube. The tubes were centrifuged for 10 min at 3000×g followed by holding at 30 min. After discarding the supernatant, the tubes were weighted again. WBC and OBC were expressed as g of water and g of oil per g of protein, respectively.

Emulsification capacity and stability

Emulsifying activity and stability of SBMP were determined using the method reported by Shilpashree et al. [31]. A total of 50 mL of 1% protein solution was mixed with 25 mL of refined soybean oil and subjected to high-shear mixing using a high-shear homogenizer (IKA ULTRA-TURRAX®) at 10000 for 5 min. The content was then transferred to two 50 mL falcon tubes and centrifuged at 1100 ×g for 5 min. The height of the emulsified layer and the total contents in the tube were measured. The emulsifying capacity was calculated as:

$$Emulsification\ capacity\ (\%) = \frac{Height\ of\ emulsified\ layer\ in\ the\ tube}{Height\ of\ total\ content\ in\ the\ tube} \times 100$$

Emulsion stability was determined by heating the emulsion at 80 °C for 30 minutes before centrifuging at 1100 ×g for 5 minutes. The ES was calculated as follows: Foaming capacity and foam stability

$$Emulsification\ stability\ (\%) = \frac{Height\ of\ emulsified\ layer\ after\ heating}{Height\ of\ total\ content\ before\ heating} \times 100$$

Foam capacity and stability

The foam capacity of the SBMP was determined by the method of Shilpashree et al. [31]. However, stirring was done additionally before blending to ensure complete mixing. The method was preceded by whipping 100 mL of 0.05 mol/L phosphate buffer (adjusted pH 7) containing 3 g protein solution. The suspension was mixed using a magnetic stirrer for 45 min, followed by mixing in an auto-mix blender (240 V AC 50 C/S, Sujata, Powermatic Plus., Bombay, India) at its maximum speed for exactly 6 min. The sample was transferred to a 500 mL graduated cylinder as quickly as possible. Foam capacity (overrun) was calculated as follows

$$\text{Foaming capacity (\%)} = \frac{(\text{B} - \text{A})}{\text{A}} \times 100$$

: Where,

A-Volume of liquid before whipping (mL);

B- Total volume (foam plus liquid) obtained immediately after whipping (mL).

Foam stability was determined as the volume of foam that remained after 30 min (30 ± 2°C) expressed as a percentage of the initial foam volume.

Buffering capacity

The buffering capacity or buffer index (molar quantity of acid or base needed to change the pH by one unit) of SKP was determined using the method reported by Mann and Malik [32]. For SBMP, 100 mL reconstituted solution containing 0.5% protein was

titrated up to pH 2 (acidification with standard acid, i.e., 0.1N HCL), and then alkalization of the same sample with standard alkali, i.e., 0.1N NaOH for each 0.5 change in pH up to 10 pH. The buffer index dB/dpH was calculated using following formula:

$$Buffering\ capacity\ (\frac{dB}{dpH}) = \frac{Volume\ of\ acid\ or\ base\ added \times normality\ of\ acid\ or\ base}{volume\ of\ sample \times pH\ change} \times 100$$

Particle size analysis

The Particle size analysis of the prepared SBMP samples was determined using a Malvern Mastersizer 3000 working on the laser diffraction technique as per the method described by Mahadev and Meena [25]. A small quantity of SBMP was dispersed in mili Q water to maintain the laser obscuration in the 12- 15% range. During measurement, non-spherical particles were considered. The refractive index and density of SBMP was taken as 1.334 and 1.30.

Estimation of antioxidant properties

Sample preparation

Five grams of SBMP were taken in 50 ml falcon tubes, and then 25 ml of 100% methanol was added. This was then centrifuged at 4000 rpm for 15 min at room temperature. Next, the supernatant was collected in another 50 ml falcon tube. Subsequently, 15 ml of both 80% and 60% methanol was added and centrifuged at the above-mentioned conditions. Finally, all the supernatant was collected in a tube and treated as a sample for the entire antioxidant assay.

2, 2′-azinobis (3- ethylbenzthiazoline-6-sulphonic acid) (ABTS)

The ABTS assay of SBMP samples were determined the method described by Salami et al. [34]. ABTS radical cation was produced by reacting ABTS stock solution (8 mM) with an equal volume of 3 mM of potassium persulphate solution and kept in dark at room temperature for 16 h. before using, stock solution was diluted with phosphate buffer (150 mM) to an absorbance of 0.70 at 734nm. Then, 100 μL of the supernatant sample was mixed with 2900 μL of working solution of ABTS. The absorbance was measured after 30 min at 734 nm in a spectrophotometer. Trolox was used as standards here and the results were mean values of triplicates.

2, 2′-diphenyl-1-picrylhydrazyl (DPPH)

The DPPH radical by added antioxidants in SBMP samples was estimated using method given by Zhang et al. [35]. 0.1mM DPPH· (prepared in 70% ethanol) was kept overnight under refrigerated condition over a magnetic stirrer for proper mixing. Four mL of DPPH was treated with 0.2 mL of supernatant extract, and control was treated with 0.2ml of methanol. The mixture was kept at room temperature for 60 mins under dark condition, followed by measuring absorbance at 517nm. The results were expressed as % antioxidant activity

$$Antioxidant\ activity\ (AA, \%) = \frac{Absorbance\ of\ control - Absorbance\ of\ sample}{Absorbance\ of\ control} \times 100$$

Ferric reducing antioxidant power (FRAP)

FRAP of SBMP was estimated using the method given by Benzie and Strain [36] with some modifications. 10mM of TPTZ (2,4,6 tri (2 pyridyl)-1,3,5 triazine), 300mM of acetate buffer and 20mM of ferric chloride solution were used to prepare FRAP reagent. 2.5ml of TPTZ stock, 25ml of acetate buffer and 2.5ml of ferric chloride solution were mixed to prepare FRAP reagent (1:10:1). 2.850 ml of FRAP reagent was mixed with 100 μl of the prepared sample extract (diluted in ethyl alcohol to 2% for antioxidant activity measurement). The absorbance was measured after exactly 4mins of mixing the sample with FRAP reagent in UV-Vis Spectrophotometer at 593nm wavelength. For the preparation of the standard curve, Trolox in different concentrations in the range of 10–100, μmol/mL was taken as standard.

Total Phenolic compounds (TPC)

Measurement of TPC wes performed using the method described by Sharma et al. [37]. Briefly, 500 μL of the supernatant sample and 2.5 mL of Folin-Ciocalteau reagent (diluted in 1:10 ratio) were taken in a test tube and kept for 5 min. Then 2 mL of sodium

carbonate solution (7.5% solution) was added and incubated in dark at the room temperature for 90 min. Then, using a spectrophotometer (UV1800, Shimadzu, Japan). , samples absorbance was measured at 765 nm The standard curve was plotted for a range of 10–100 μg/mL and results were expressed as μg gallic acid equivalent/mL The TPC analysis powder results were expressed as micrograms of gallic acid equivalent (GAE). Total flavonoid content

Total flavonoid content was neasured by the method suggested by Sharma et al., [37]. Two mL of each SBMP supernatant and aluminium chloride solution (2%, w/v) was taken in a test tube, kept undisturbed for incubation at room temperature for 1 h and absorbance was measurement at 420 nm. Quercetin was used to plot the standard curve at a range of 1–10 μg/mL. The results were expressed as μg /mL.

Fatty acid profiling of SBMP

Fatty Acid Methyl Ester (FAME) derivatization

FAME derivatization was carried out according to the procedure of ISO 15884 [38]. Approximately 20 mg of the extracted fat of SBMP sample was weighed in a 15 ml centrifuge tube. The sample was dissolved in 5 ml of hexane and was mixed well. Next, 0.2 ml of the transesterification reagent was added to the falcon tubes and capped. Contents of the tube were mixed vigorously using a vortex mixer for 1 min. After an additional reaction time of 5 min, 0.5 g of solid sodium hydrogen sulphate was added, and again it was mixed. Test tubes with the test portion were placed in the centrifuge and centrifuged at 350 g for 3 min at room temperature. After centrifugation, an aliquot was taken from the obtained clear supernatant of the test portion for the gas-liquid chromatographic analysis or the ester solution was decanted and stored in vials. Preferably, it was kept in a deep-freezer for several days, and precautions should be taken to avoid losses due to the volatility of methyl esters of milk fat fatty acids.

Gas chromatography analysis

One μl of methyl ester prepared FAME samples were injected using autosampler (AOC-20i) in a split less mode in a GC-FID system coupled with a a Flame ionization detector. Chromatographic separation of FAME was performed on SPTM-2560 (Fused Silica Capillary Column; 100 m X 0.25 mm i.d. X 0.20 μm film thickness, Supleco). Nitrogen was used as carrier gas with a column flow of 0.8 ml/min; the oven program started with an initial temperature of 140 °C (hold time 5 min), which was ramped to 240 °C at the rate of 3 °C /min (hold time 15 min). The temperature of the injector and ion source was maintained at 250 °C. The fragmentation spectra of 37 standard FAME mixes (Supelco) were matched with a reference chromatogram.

GC conditions

Column: SPTM-2560 (Fused Silica Capillary Column; 100 m X 0.25 mm i.d. X 0.20 μm film thickness, Supelco)

Oven temperature: 140°C

Injection temperature: 250°C

Column flow: 0.8 ml/min

Quantification of FAME samples

FAME samples were run at GC conditions, and fragmentation spectra of analyte molecules were matched with a reference standard (Supelco 37 component FAME mix, Sigma Aldrich India.), and Quantification of the sample was done by area normalization method in which amount of sample components are estimated based on the area/height of the peaks of the components relative to the total area multiplied by 100 (% composition).
